# Editorial: Comprehensive insights into microbial infection: from pathogenesis to therapeutic solutions

**DOI:** 10.3389/fcimb.2026.1842438

**Published:** 2026-04-14

**Authors:** Rahul Kumar Maurya, Pooja Agarwal, Ashutosh Kumar, Suman Bharti

**Affiliations:** 1 Division of Pulmonary and Critical Care Medicine, Department of Medicine, Washington University in St. Louis, St. Louis, MO, United States; 2 Warwick Medical School, University of Warwick, Coventry, United Kingdom; 3 Department of Internal Medicine, Cardiovascular Division, Washington University School of Medicine, St. Louis, MI, United States; 4 Department of Medicine, Washington University School of Medicine, St. Louis, MI, United States

**Keywords:** disease progression, immune responses, microbial infections, pathogenesis, therapeutic strategies

Microbial infections caused by bacteria and viruses continue to represent a significant global health burden and affecting millions of individuals each year. Despite decades of research and remarkable advances in biomedical and clinical medicine, infectious diseases remain a major cause of morbidity and mortality worldwide. A deeper understanding of complexity of microbial pathogenesis and host immune responses are essential for identifying novel therapeutic targets. In recent years, pathogens have evolved diverse strategies to evade host immune defenses while host immune system also simultaneously deploys complex responses to detect and eliminate invading microorganisms. Disruption of this delicate balance can lead to severe disease outcomes, chronic infections, or immune dysregulation. Continued investigation into these interactions that integrates clinical observations with mechanistic insights is essential for improving diagnostic approaches against infectious diseases.

**Figure 1:**
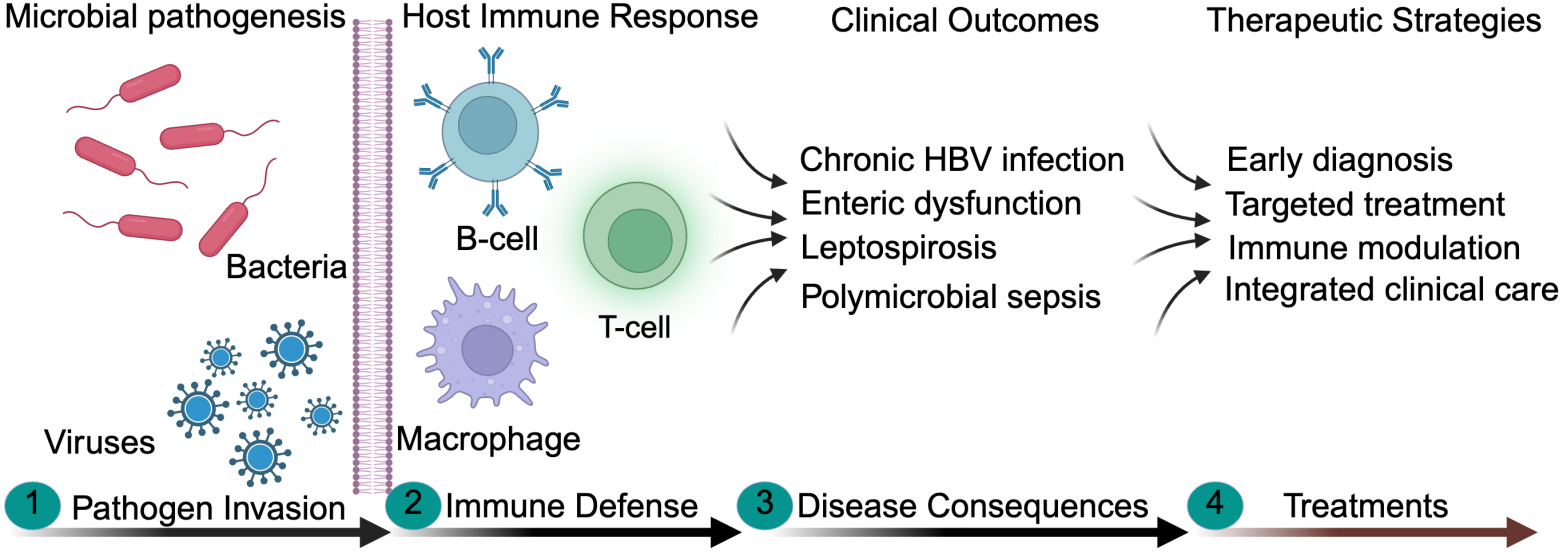
This schematic illustrates the sequential events following microbial invasion and their clinical implications. (1). Bacterial and viral pathogens enter host tissues and initiate infection. (2) Subsequent innate immune components activate antigen presenting cells and promotes immune signaling. (3) Activation of adaptive immunity including T- cell responses promote cytokine release and inflammatory responses. (4) These dysregulated immune responses influence disease progression, while targeted therapeutic interventions aim to restore immune balance and improve clinical outcomes.

The goal of this Research Topic was organized to provide a platform for presenting recent advances in microbial pathogenesis, host-pathogen interactions, and innovative therapeutic approaches. This Research Topic highlights interdisciplinary research that bridges diverse aspects of biological discoveries with translational and clinical applications. The articles included in this Research Topic collectively address how microbial infections influence disease severity, immune dysregulation during infection, clinical outcomes, and disease progression (**Figure 1**).

One of the studies in this Research Topic investigates clinical predictors associated with severe leptospirosis, a reemerging zoonotic infection with diverse clinical manifestations. The authors conducted a retrospective case control study examining the patients diagnosed with leptospirosis in the Transcarpathian region of Ukraine. Their findings identified oliguria as a key independent clinical marker associated with severe disease and mortality which emphasizing the early recognition may improve patient management/outcomes by integrating clinical observations with epidemiological surveillance (Petakh and Kamyshnyi). Another contribution examines the complex immunological interplay between T and B cells during chronic hepatitis B virus (HBV) infection, a disease affecting millions of people worldwide. Chronic HBV infection is characterized by persistent viral replication and immune dysregulation that contributes to disease progression. The study highlighted how immune dysfunction contributes to viral persistence (Yu et al.). While, the authors also discuss emerging immunotherapeutic strategies, including therapeutic vaccines, immune checkpoint inhibitors, T cell-based therapies that aim to restore antiviral immunity. These insights contribute a deeper understanding of adaptive immune mechanisms in chronic viral infections. The ongoing evolution of viral pathogens further illustrates the importance of understanding host immune responses during infection. In the context of the COVID-19 pandemic, one study investigates the leukocyte immune responses to ancestral severe acute respiratory syndrome coronavirus 2 (SARS-CoV-2) and its variants. By analyzing hematological data from COVID-19 patients, the authors demonstrate that immune responses vary depending on the viral variant with distinct patterns of suppression and activation of leukocyte that associated with disease severity (Wu et al.).

These findings highlight the dynamic nature of host immune responses to viral evolution. Another study included in this Research Topic investigates the role of enteric pathogens in environmental enteric dysfunction (EED) in malnourished adults living in Bangladesh. EED is a subclinical intestinal disorder associated with chronic exposure to enteric pathogens and is frequently linked to poor sanitation and malnutrition. The results revealed that specific *Escherichia coli* (*E. coli*) pathotypes are associated with markers of intestinal inflammation (Sthity et al.) and gut permeability which provide the important insights of pathogen driven intestinal pathology in vulnerable populations. Finally, an experimental study examining the cellular and microbial dynamics of polymicrobial sepsis using a murine model. Sepsis represents a life-threatening condition characterized by dysregulated immune responses to infection and remains a major cause of mortality worldwide. Using multiplexed longitudinal analyses, the authors describe how immune responses shift from a hyperinflammatory state to immunosuppression and further identified key immune cell populations involved in disease progression (Peacock et al.). Experimental models like this work provides valuable insights into the complex immunopathology of sepsis and offer animal models for identifying biomarkers and infection dynamics.

Collectively, the studies included in this Research Topic provide a diverse perspective on microbial pathogenesis, host immune responses, and potential therapeutic strategies. Continued research in this field advances our understanding of microbial pathogenesis and their impact on human health.

